# Efficacy of surface disinfectant cleaners against emerging highly resistant gram-negative bacteria

**DOI:** 10.1186/1471-2334-14-292

**Published:** 2014-05-28

**Authors:** Mirja Reichel, Anastasija Schlicht, Christiane Ostermeyer, Günter Kampf

**Affiliations:** 1 Bode Science Center, Bode Chemie GmbH, Melanchthonstr. 27, 22525 Hamburg, Germany; 2 Labor L + S AG, Mangelsfeld 4, 5, 6, 97708, Bad Bocklet-Groβenbrach, Germany; 3 Microbiology, Bode Chemie GmbH, Melanchthonstr. 27, 22525 Hamburg, Germany; 4 Institut für Hygiene und Umweltmedizin, Ernst-Moritz-Arndt Universität Greifswald, Walther-Rathenau-Straβe 49a, 17475 Greifswald, Germany

**Keywords:** Surface disinfection cleaner, Gram-negative bacteria, Multidrug resistance, Pan-drug resistance

## Abstract

**Background:**

Worldwide, the emergence of multidrug-resistant gram-negative bacteria is a clinical problem. Surface disinfectant cleaners (SDCs) that are effective against these bacteria are needed for use in high risk areas around patients and on multi-touch surfaces. We determined the efficacy of several SDCs against clinically relevant bacterial species with and without common types of multidrug resistance.

**Methods:**

Bacteria species used were ATCC strains; clinical isolates classified as antibiotic-susceptible; and multi-resistant clinical isolates from *Klebsiella oxytoca*, *Klebsiella pneumoniae*, and *Serratia marcescens* (all OXA-48 and KPC-2)*; Acinetobacter baumannii* (OXA-23); *Pseudomonas aeruginosa* (VIM-1)*;* and *Achromobacter xylosoxidans* (ATCC strain). Experiments were carried out according to EN 13727:2012 in quadruplicate under dirty conditions. The five evaluated SDCs were based on alcohol and an amphoteric substance (AAS), an oxygen-releaser (OR), surface-active substances (SAS), or surface-active-substances plus aldehydes (SASA; two formulations). Bactericidal concentrations of SDCs were determined at two different contact times. Efficacy was defined as a log_10_ ≥ 5 reduction in bacterial cell count.

**Results:**

SDCs based on AAS, OR, and SAS were effective against all six species irrespective of the degree of multi-resistance. The SASA formulations were effective against the bacteria irrespective of degree of multi-resistance except for one of the four *P. aeruginosa* isolates (VIM-1). We found no general correlation between SDC efficacy and degree of antibiotic resistance.

**Conclusions:**

SDCs were generally effective against gram-negative bacteria with and without multidrug resistance. SDCs are therefore suitable for surface disinfection in the immediate proximity of patients. Single bacterial isolates, however, might have reduced susceptibility to selected biocidal agents.

## Background

Healthcare-associated infections, especially with multidrug-resistant gram-negative bacteria (MRGN) are an emerging problem in infection control [1]. MRGN are responsible for serious infections and have a significant impact on morbidity and mortality [2]. The spread of these organisms as well as the spread of resistance genes is an emerging public health issue [3,4]. Only limited therapeutic options are available and finding effective and suitable antibiotic drugs to treat infections can be difficult.

The main influence on the development of antibiotic drug resistance is antibiotic use, both the total amount of antibiotics used and the distribution of antimicrobial classes. For example, data from Germany show that despite a stable total amount of antibiotics used between 2001 and 2008, carbapenem use doubled. This was associated with an increase in carbapenem-resistant *Klebsiella pneumonia*, carbapenemase-producing bacteria and imipenem-resistant *Acinetobacter baumannii*[5].

Once resistance is established, MRGN spread, especially with global travel [6]. Reports of MRGN outbreaks are abundant in the literature [7,8]. Recent data from Russia show that extensively drug-resistant *Pseudomonas aeruginosa* spread rapidly throughout Russia and into Belarus and Kazakhstan [9]. The damage of MRGN must be limited by preventing transmission.

This strategy is reflected in a 2012 recommendation from the Commission for Hospital Hygiene and Infectious Disease Prevention (KRINKO) at the Robert Koch-Institute in Germany for handling patients infected or colonized with MRGN. In 2014, the ESCMID also published a guideline on infection control measures MRGN in hospitalized patients [10]. In contrast to the more epidemiologically based approach of Magiorakos et al. [11], the German recommendation focused on the clinical relevance of the resistance pattern. For individual pathogens, the most important antibiotics for treating severe infections were determined by resistance pattern (acylureidopenicillins, third-generation and fourth-generation cephalosporins, carbapenems, and fluoroquinolones). According to the KRINKO definition, 3MRGN are resistant to three of these four classes; 4MRGN are gram-negative microorganisms resistant to all four classes and include pan-resistant microorganisms. The recent ESCMID guideline does not distinguish different resistance patterns within MRGN [10].

A key factor in prevention of MRGN infections is the consequent implementation and compliance with effective hygiene measures [12,13] which is also described in the ESCMID guideline [10]. Targeted surface disinfection is a major measure of standard infection control. The surface disinfectants must be effective against the targeted pathogens. Surfaces near patients and high-touch surfaces must be effectively disinfected.

Surface disinfectant cleaners (SDCs) that are often used for this purpose and can be used in the immediate proximity of patients are usually based on surface-active ingredients such as quaternary ammonium compounds (QACs). Some data indicate that adaptation or resistance to QACs can develop and QACs have greater activity against gram-positive bacteria compared to gram-negative bacteria [14,15]. Therefore, we determined the efficacy of several common SDCs against clinically relevant bacterial species with and without common types of multidrug resistance.

## Methods

### Test products

Five SDCs were tested at the concentration which is listed to be bactericidal using standard test bacteria (Table 1). Bacillol 30 foam, based on alcohols and an amphoteric substance (AAS) was tested undiluted for 30 and 60 s; Mikrobac forte, based on surface-active substances (SAS) was tested at 0.5% for 30 and 60 min; Dismozon plus, based on an oxygen-releaser (OR), was tested at 0.4% for 30 and 60 min; Kohrsolin extra, based on surface-active substances and an aldehyde (SASA1), was tested at 0.25% for 30 and 60 min; and Kohrsolin FF, based on surface-active substances and an aldehyde (SASA2), was tested at 0.5% for 30 and 60 min. All SDCs were manufactured by Bode Chemie GmbH, Hamburg, Germany. Products were blinded for the investigation.

**Table 1:** **Surface disinfectant cleaners, their active ingredients and bactericidal concentration as listed by the Commission for disinfectants **[53]

**Product**	**Abbreviation**	**Active ingredients**	**Bactericidal concentration (exposure time)**
Bacillol® 30 foam	AAS	Ethanol; propan-2-ol; propan-1-ol; N-alkyl-aminopropylglycine	Undiluted (30 s)
Mikrobac® forte	SAS	Benzyl-C12-18- alkyldimethylammonium chlorides; N-(3-aminopropyl)-N dodecylpropane- 1,3-diamine	0.5% (1 h)
Dismozon® plus	OR	Magnesium monoperoxyphthalate hexahydrate	0.4% (1 h)
Kohrsolin® extra	SASA1	(Ethylenedioxy) dimethanol; Glutaral; Didecyldimethylammonium chloride	0.25% (1 h)
Kohrsolin® FF	SASA2	Glutaral; Benzyl-C12-18-alkyldimethylammonium chlorides; Didecyldimethylammonium chloride	0.5% (1 h)

### Tested bacterial species

Bacterial species were selected because they are specifically mentioned in the German and the European recommendation (*K. pneumoniae*, *P. aeruginosa*, *A. baumannii*) [10,13], or because they have been described before to be potentially less susceptible to selected biocidal agents of SDCs (*Serratia marcescens*, *Klebsiella oxytoca*, *Achromobacter xylosoxidans*) [16-18]. They were used as: *S. marcescens* (ATCC 14756 and four clinical isolates: antibiotic-susceptible (“0MRGN”), 3MRGN, 4MRGN OXA-48 and 4MRGN KPC-2); *K. pneumoniae* (ATCC 10031 and four clinical isolates: antibiotic-susceptible, 3MRGN, 4MRGN OXA-48 and 4MRGN KPC-2); *K. oxytoca* (ATCC 700324 and four clinical isolates: antibiotic-susceptible, 3MRGN, 4MRGN OXA-48 and 4MRGN KPC-2); *P. aeruginosa* (ATCC 15442 and three clinical isolates: antibiotic-susceptible, 3MRGN and 4MRGN VIM-1); *A. baumannii* (ATCC 19606 and two clinical isolates: antibiotic-susceptible and 4MRGN OXA-23); and *A. xylosoxidans* (ATCC 27061). The eight 4MRGN isolates were from the National Reference Laboratory for multidrug-resistant gram-negative bacteria, Ruhr University Bochum, Germany. The five antibiotic-susceptible isolates and four 3MRGN isolates were kindly provided by the Laboratory Fenner, Hamburg, Germany.

### Determination of bactericidal activity

All experiments were carried out at Labor L + S AG**,** Bad Bocklet, Germany, according to EN13727:2012 which is the suspension test to determine the spectrum of bactericidal activity of disinfectants used in human medicine [19]. All tested products are used as disinfectant cleaners so dirty conditions with an interfering substance of 0.3% bovine albumin and 0.3% sheep erythrocytes were used. Each experiment was carried out in quadruplicate on different test days and by different investigators.

From a second subculture on agar plates, a test suspension was adjusted to 1.5–5.0 × 10^8^ colony forming units (cfu) per ml. To 1 ml test suspension, 1 ml interfering substance with 3% bovine albumin and 3% sheep erythrocytes was added and mixed. The mixture was placed in a water bath (20 ± 1°C) for 2 min and 8 ml of tested product was added. After mixing, the tube was placed in the water bath for the indicated time, mixed again before the end of incubation, and 1 ml added to a tube with 8 ml neutralizing broth (3.0% Tween 80, 3.0% saponin, 0.1% histidine, 0.1% cysteine in tryptic soy broth) and 1 ml water. After mixing, the tube was placed in the water bath for 5 min (for 30 or 60 min contact times) or 10 s (for 30 or 60 s contact times). Neutralizing agents were validated for all SDCs using all species as ATCC strains. At the end of neutralization, the sample was mixed, and diluted 1:10 with neutralizing broth. 0.5 ml of sample without dilution was plated in quadruplicate and 0.5 ml of each dilution step in duplicate on TSA containing neutralizers (Biomérieux, Nürtingen, Germany and heipha Dr. Müller GmbH, Eppelheim, Germany). Plates were incubated at 37 ± 1°C for 48 h and colonies per plate counted. All plates from a dilution step with <330 cfu were used to calculate the number of cfu/mL in a sample of disinfectant, test organism, and interfering substance. Data were converted to log_10_ scale. Bacterial reduction was calculated as viable colonies before exposure to a disinfectant minus viable colonies after exposure. The EN13727 requirement for bactericidal activity is a log_10_ reduction ≥5 within the chosen contact time. Controls for experimental conditions and neutralizer, and dilution neutralization validation were carried out according to EN 13727.

### Data presentation

When all four experiments per product, test organism and time indicated a log_10_ reduction ≥5, the lowest log_10_ reduction of the four results is presented. If the four experiments indicated a log_10_ reduction <5, mean and standard deviation were calculated. A general correlation between efficacy of a SDC and bacterial antibiotic-resistance status was assumed if a SDC was less effective against a 4MRGN compared to a 3MRGN and if it was at the same time less effective against a 3MRGN compared to a 0MRGN.

## Results

All AAS, SAS, and OR products were effective (≥5 log_10_-reduction) against all tested species, the ATCC strains and the clinical isolates with and without multidrug-resistance (Tables 2 and 3). No efficacy gap was observed among the products against the tested gram-negative strains. SASA products were also comprehensively effective against *S. marcescens*, *K. pneumonia*, *K. oxytoca*, *A. baumannii* and *A. xylosoxidans*, both ATCC strains and clinical isolates with and without multidrug-resistance. They were also effective against *P. aeruginosa* ATCC 15442, 0MRGN and 3MRGN but were not sufficiently effective against 4MRGN VIM-1 with a mean log_10_-reduction of 1.54–3.45 (Table 3). No general correlation was seen between efficacy of all five SDC and bacterial antibiotic-resistance status with five of six bacterial species (25 possible correlations). It was also not seen for three SDCs with *P. aeruginosa* (3 possible correlations). It was partly seen for 2 SASA products with *P. aeruginosa* but only for the comparison of 4MRGN versus 3MRGN and not for the comparison 3MRGN versus 0MRGN.

**Table 2:** **Log**_**10**_**-reduction of cell counts by five surface disinfectant cleaners against emerging multidrug-resistant enterobacteriaceae and corresponding ATCC strains**

**Species**	**Isolate/strain**	**Product (concentration)**	**Contact time**	**Log**_**10**_**-reduction**
*Serratia marcescens*	ATCC 14756	AAS (undiluted)	30 and 60 s	> 5.47
		SAS (0.5%)	30 and 60 min	
		OR (0.4%)		
		SASA1 (0.25%)		
		SASA2 (0.5%)		
	0MRGN	AAS (undiluted)	30 and 60 s	> 5.37
		SAS (0.5%)	30 and 60 min	
		OR (0.4%)		
		SASA1 (0.25%)		
		SASA2 (0.5%)		
	3MRGN	AAS (undiluted)	30 and 60 s	> 5.17
		SAS (0.5%)	30 and 60 min	
		OR (0.4%)		
		SASA1 (0.25%)		
		SASA2 (0.5%)		
	4MRGN OXA-48	AAS (undiluted)	30 and 60 s	> 5.14
		SAS (0.5%)	30 and 60 min	
		OR (0.4%)		
		SASA1 (0.25%)		
		SASA2 (0.5%)		
	4MRGN KPC-2	AAS (undiluted)	30 and 60 s	> 5.09
		SAS (0.5%)	30 and 60 min	
		OR (0.4%)		
		SASA1 (0.25%)		
		SASA2 (0.5%)		
*Klebsiella pneumoniae*	ATCC 10031	AAS (undiluted)	30 and 60 s	>5.34
		SAS (0.5%)	30 and 60 min	
		OR (0.4%)		
		SASA1 (0.25%)		
		SASA2 (0.5%)		
	0MRGN	AAS (undiluted)	30 and 60 s	>5.08
		SAS (0.5%)	30 and 60 min	
		OR (0.4%)		
		SASA1 (0.25%)		
		SASA2 (0.5%)		
	3MRGN	AAS (undiluted)	30 and 60 s	> 5.09
		SAS (0.5%)	30 and 60 min	
		OR (0.4%)		
		SASA1 (0.25%)		
		SASA2 (0.5%)		
	4MRGN OXA-48	AAS (undiluted)	30 and 60 s	>5.38
		SAS (0.5%)	30 and 60 min	
		OR (0.4%)		
		SASA1 (0.25%)		
		SASA2 (0.5%)		
	4MRGN KPC-2	AAS (undiluted)	30 and 60 s	>5.34
		SAS (0.5%)	30 and 60 min	
		OR (0.4%)		
		SASA1 (0.25%)		
		SASA2 (0.5%)		
*Klebsiella oxytoca*	ATCC 700324	AAS (undiluted)	30 and 60 s	>5.14
		SAS (0.5%)	30 and 60 min	
		OR (0.4%)		
		SASA1 (0.25%)		
		SASA2 (0.5%)		
	0MRGN	AAS (undiluted)	30 and 60 s	>5.36
		SAS (0.5%)	30 and 60 min	
		OR (0.4%)		
		SASA1 (0.25%)		
		SASA2 (0.5%)		
	3MRGN	AAS (undiluted)	30 and 60 s	>5.31
		SAS (0.5%)	30 and 60 min	
		OR (0.4%)		
		SASA1 (0.25%)		
		SASA2 (0.5%)		
	4MRGN OXA-48	AAS (undiluted)	30 and 60 s	>5.31
		SAS (0.5%)	30 and 60 min	
		OR (0.4%)		
		SASA1 (0.25%)		
		SASA2 (0.5%)		
	4MRGN KPC-2	AAS (undiluted)	30 and 60 s	>5.11
		SAS (0.5%)	30 and 60 min	
		OR (0.4%)		
		SASA1 (0.25%)		
		SASA2 (0.5%)		

Experiments were carried out in quadruplicate under dirty conditions; lowest log_10_-reduction values are shown.

**Table 3:** **Log**_**10 **_**reduction of cells counts by five surface disinfectant cleaners against emerging multidrug-resistant non-fermenting bacteria and corresponding ATCC strains**

**Species**	**Isolate/strain**	**Product (concentration)**	**Contact time**	**Log**_**10**_**-reduction**
*Pseudomonas aeruginosa*	ATCC 15442	AAS (undiluted)	30 and 60 s	>5.28
		SAS (0.5%)	30 and 60 min	
		OR (0.4%)		
		SASA1 (0.25%)		
		SASA2 (0.5%)		
	0MRGN	AAS (undiluted)	30 and 60 s	>5.14
		SAS (0.5%)	30 and 60 min	
		OR (0.4%)		
		SASA1 (0.25%)		
		SASA2 (0.5%)		
	3MRGN	AAS (undiluted)	30 and 60 s	>5.06
		SAS (0.5%)	30 and 60 min	
		OR (0.4%)		
		SASA1 (0.25%)		
		SASA2 (0.5%)		
	4MRGN VIM-1	AAS (undiluted)	30 s and 60 s	>5.45
		SAS (0.5%)	30 and 60 min	
		OR (0.4%)		
		SASA1 (0.25%)	30 min	1.54 ± 0.91
			60 min	1.76 ± 0.94
		SASA2 (0.5%)	30 min	3.04 ± 0.73
			60 min	3.45 ± 0.55
*Acinetobacter baumannii*	ATCC 19606	AAS (undiluted)	30 and 60 s	>5.20
		SAS (0.5%)	30 and 60 min	
		OR (0.4%)		
		SASA1 (0.25%)		
		SASA2 (0.5%)		
	0MRGN	AAS (undiluted)	30 and 60 s	>5.09
		SAS (0.5%)	30 and 60 min	
		OR (0.4%)		
		SASA1 (0.25%)		
		SASA2 (0.5%)		
	4MRGN OXA-23	AAS (undiluted)	30 and 60 s	>5.14
		SAS (0.5%)	30 and 60 min	
		OR (0.4%)		
		SASA1 (0.25%)		
		SASA2 (0.5%)		
*Achromobacter xylosoxidans*	ATCC 27061	AAS (undiluted)	30 and 60 s	>5.13
		SAS (0.5%)	30 and 60 min	
		OR (0.4%)		
		SASA1 (0.25%)		
		SASA2 (0.5%)		

Experiments were carried out in quadruplicate under dirty conditions; lowest log_10_ reduction values or mean and standard deviation are shown.

## Discussion

All SDCs containing SAS were generally effective against MRGN. Literature reports on SAS efficacy are conflicting. Some studies found that SAS are not sufficiently effective against gram-negative bacteria or that efficacy is lower against gram-negative than against gram-positive bacteria [14,20-23]*.* Other studies found that SAS are effective [24,25]. Outbreaks of contaminated SAS disinfectant solutions have been reported [26-28]. Some strains, particularly biofilm-forming species, survive or even multiply in SAS disinfectants at concentrations at which they are normally used and this can result in infections such as septicemia [29,30]. Our results did not support the hypothesis that SAS-containing products are insufficiently effective against gram-negative bacteria. We found that SAS products were highly effective against multiple clinically relevant gram-negative microorganisms.

The link between lower susceptibility to SAS and antibiotic resistance is not conclusively established [31,32]. No resistance breakpoints have been defined for biocides so defining resistance to these compounds is difficult. In addition, reversible adaptations to an active ingredient versus stable resistance must be distinguished [33,34]. SAS are used for multiple applications, e.g., in the cosmetic, pharmaceutical, and food industries. Adaptation and resistance have been shown for different species. For gram-negative bacteria, cross-resistance to different antibiotics and to different types of SAS, and selection for antibiotic-resistant strains has been found [35,36]. Antibiotic resistance and resistance to biocides can have the same molecular mechanisms, although biocides generally show a broader activity because their mode of action is nonspecific [37-39]. The association between antibiotic-resistance and biocide-resistance seen in gram-negative bacteria can be explained by a link between the genes for both resistance mechanisms [40]. Of note, studies on resistance are often carried out with concentrations of surface disinfectants lower than what is normally used. To avoid concentrations of SDC that are sublethal to bacteria, recommended concentrations and contact times must be known and used.

We did not find a general correlation between antibiotic-resistance patterns and susceptibility to SAS-containing products, consistent with earlier reports [41]. Only a single 4MRGN *P. aeruginosa* isolate had lower susceptibility to the two aldehyde-containing SAS products. Nonetheless, the products were highly effective against three other *P. aeruginosa* isolates indicating that reduced susceptibility was strain-specific. Individual clinical strains can show reduced susceptibility to aldehyde [42,43]; especially for some *P. aeruginosa* strains, published data are available on the lower efficacy of aldehyde-containing products [44-46]. We do not know the underlying mechanism of the reduced susceptibility of the isolate in our study. However, determining the molecular mechanism of the lowered susceptibility would be interesting and help to better understand our findings.

We found that products based on an OR compound or AAS were highly effective. A few studies with alcohol-based hand rubs revealed that they are effective against multidrug-resistant bacterial isolates including multidrug-resistant *Mycobacterium tuberculosis*[47,48]. No studies have found that OR compounds used on surfaces are not fully effective against bacteria. Both OR and AAS are considered to have a nonspecific mechanism of action [49] and are volatile (alcohol) or degradable (OR compounds). Therefore, acquired bacterial resistance to OR or AAS products is very unlikely.

A limitation of this study for clinical practice is experiments were carried out with bacteria in suspension and not under practical conditions. Suspension tests are the first-choice method for studying the spectrum of antimicrobial activity of disinfectants [50]. Testing the effectiveness of the formulations under practical conditions would also be interesting. In Europe, a test method is currently being developed to determine both the efficacy of surface disinfectants on a contaminated test field and the potential to spread of bacteria by wiping to non-contaminated surfaces [51]. Future research could determine the effectiveness of SDCs tested under practical conditions.

Recent data from Germany show that the percentage of MRGN in a hospital exceeded nosocomial infection rates by MRSA and VRE (8% vs. 2% for MRSA and VRE) [52]. The main reason is that once multidrug-resistance is established, the spread of resistant strains is inevitable. Currently, no effective sanitation methods exist against MRGN carriers. Even with contact precautions in patient care, standard hygiene measures are still essential for preventing of transmission of MRGN. Targeted surface cleaning and disinfection is an element of standard hygiene in hospitals.

## Conclusions

Our data showed that SDCs with different active ingredients were generally effective against a variety of emerging multidrug-resistant bacterial species.

## Competing interests

MR, CO and GK are employed by BODE Chemie GmbH, Hamburg, Germany.

## Authors’ contribution

MR and GK designed the study. AS was responsible for the performing all experiments. AS and MR analyzed the data. MR and GK drafted the manuscript; AS and CO critically revised the manuscript. All authors approved the final version of the manuscript.

## Pre-publication history

The pre-publication history for this paper can be accessed here:

http://www.biomedcentral.com/1471-2334/14/292/prepub
